# Supplementary material for: The role of FraI in cell–cell communication and differentiation in the hormogonia-forming cyanobacterium Nostoc punctiforme
Source: mSphere. 2024 Jul 22;9(8):e00510-24. doi: 10.1128/msphere.00510-24 (PMC11351039; doi:10.1128/msphere.00510-24)
Supplement: Supplemental material — Table S1, Video S1 legend, and Fig. S1–S7. [file msphere.00510-24-s0001.pdf]

Supplementary Materials for:

The role of FraI in cell-cell communication and differentiation in the hormogonia-forming cyanobacterium Nostoc punctiforme

Ana Janović [1], Iris Maldener [1,*], Claudia Menzel [1], Gabriel A. Parrett [2], Douglas D. Risser [2,*]

[1] Interfaculty Institute of Microbiology and Infection Medicine Tübingen, Organismic Interactions, University of Tübingen, Auf der Morgenstelle 28, 72076 Tübingen, Germany

[2] Department of Biology, University of Colorado, Colorado Springs, USA

[*] Correspondence: drisser@uccs.edu; iris.maldener@uni-tuebingen.de

Table S1. - Strains, plasmids and primers used in this study.

strain name	description
N. punctiforme ATCC29133	wild type
UCCS 103	In-frame deletion of *fraI* (Δ*fraI*)
UCCS 113	Native *fraI* allele replaced with *fraI-gfpuv*
UCTN1	Tn5 insertion at 225 bp of fraI coding-region

plasmid name	description
pDDR558	Suicide vector for in-frame deletion of *fraI* (Npun_F4142)
pGAP109	Suicide vector for replacement of native *fraI* allele with *fraI-gfpuv*

Primer name	sequence
NpF4142-5'-F	atataggatccGGAAACGCACTGATTGAAAAC
NpF4142-5'-R	cactttattacctttgCAATAGAAACATAGATTAACTCTC
NpF4142-3'-F	ctatgtttctattgCAAAGGTAATAAAGTGAGAGAG
NpF4142-3'-R	atatagagctcAAAGGGTAAACTGCCAGAG
NpF4142-gfp-5'-R	atatacccgggACCTTTGAACACGCAAGAAC
NpF4142-gfp-3'-F	atataactagtTAAAGTGAGAGAGAAGTCAG

Video S1. Time lapse microscopy of the wild type and Δ*fraI* mutant.

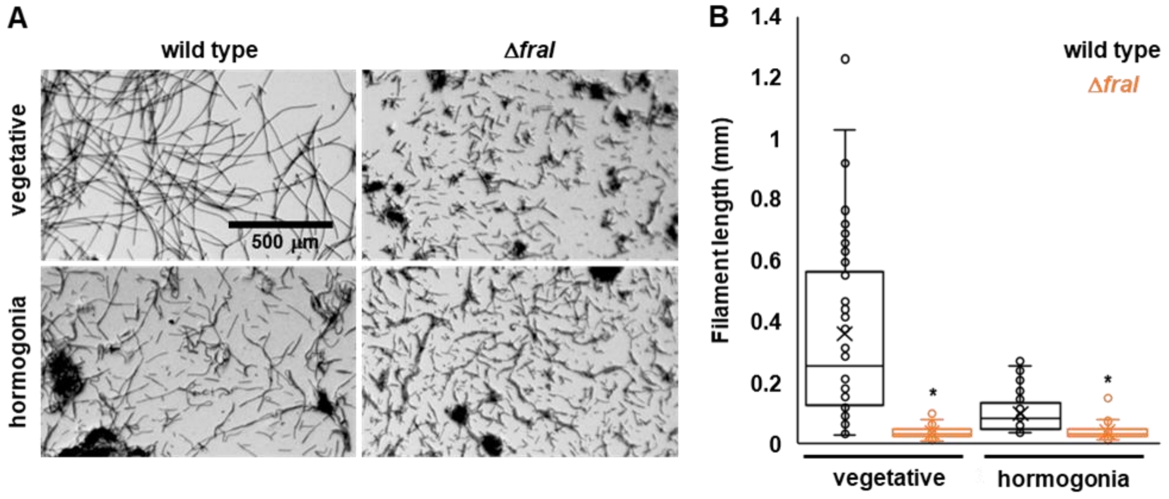

Figure S1. Quantification of filament length in the Δ*fraI* mutant. **(A)** Micrographs of vegetative and hormogonium filaments from liquid cultures of the wild type and Δ*fraI* strain. **(B)** Quantification of filament length. Box plots display the length of a total of 60 filaments, 20 from each of three biological replicates for each strain and filament type. * = p-value <0.0001 for students T-test comparing the average of the three biological replicates between the wild type and Δ*fraI* strain for either vegetative or hormogonium filaments.

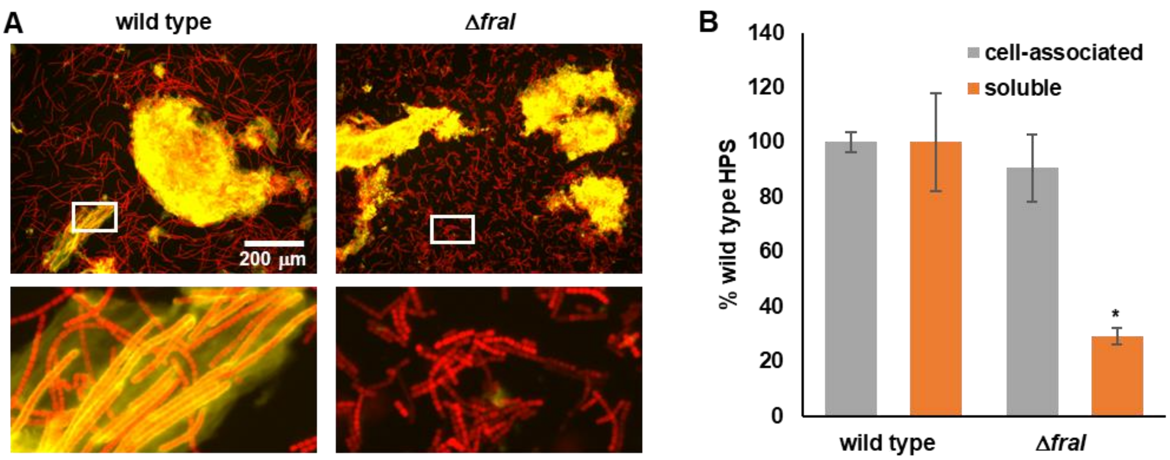

Figure S2. Lectin-based analysis of HPS in the Δ*fraI* mutant. **(A)** Fluorescence micrographs of hormogonium filaments from liquid cultures of the wild type and Δ*fraI* strain. Red = autofluorescence, yellow = HPS stained with UEA-fluorescein. **(B)** Quantification of cell-associated and soluble HPS by lectin analysis. * = p-value <0.01 for students T-test comparing the average of the three biological replicates between the wild type and Δ*fraI* strain for either cell-associated or soluble HPS.

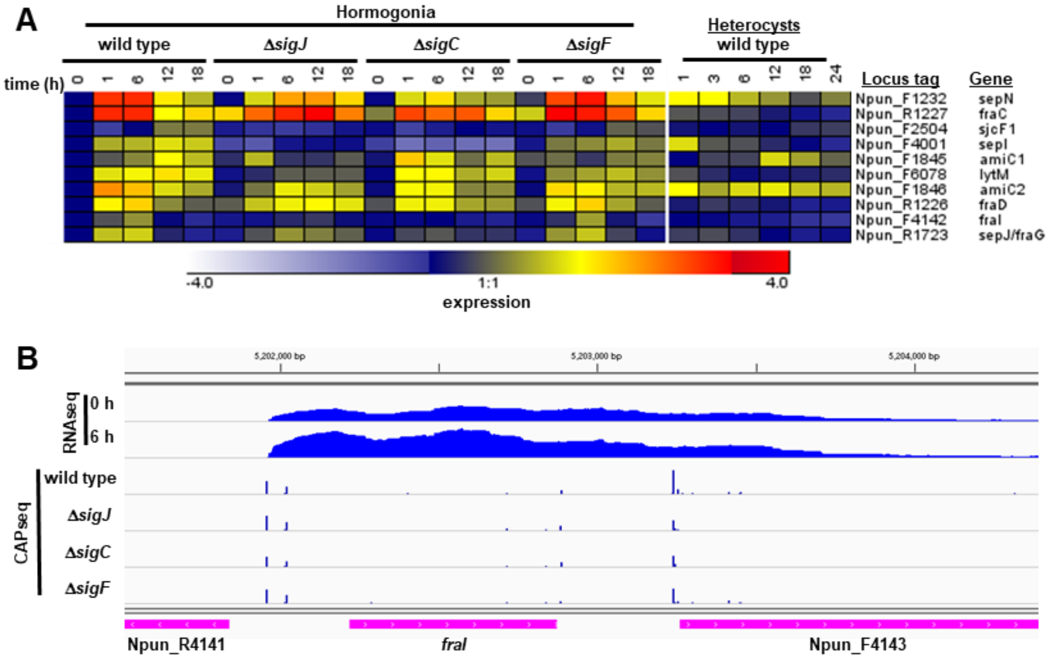

Figure S3. Transcription of septal junction genes in developing hormogonia and heterocysts in *N. punctiforme* **(A)** Heat maps depicting the expression of *fraI* and other septal junction genes in developing hormogonia of the wild-type and hormogonium-specific sigma factor mutants 0-18 h post hormogonium induction, or in developing heterocysts 0-120 h post heterocyst induction. Expression = Log2(experimental strain and time point/wild type t=0). **(B)** Read map coverage of the *fraI* locus from RNAseq (0 or 6 h post hormogonium induction) and Cappable-Seq (6 h post hormogonium induction) data for various strains as indicated.

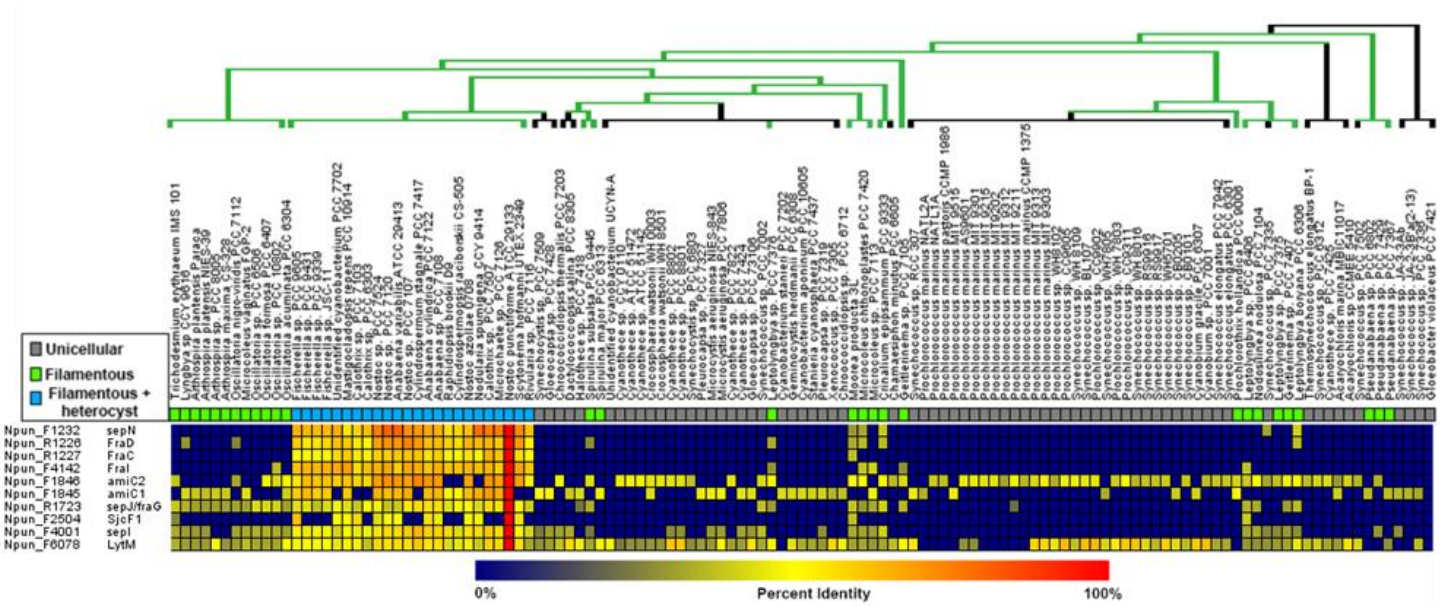

Figure S4. Evolutionary conservation of genes encoding septal junction proteins in cyanobacteria. Heat map depicting the percent identity for orthologs of *N. punctiforme* septal junction genes in cyanobacteria, derived from data reported by Cho *et al*.[1]. Species organization and phylogenetic tree based on the phylogeny reported by Shih *et al*.[2] , but depicting the finding, as reported by Schirrmeister *et al*.[3], that most extant cyanobacteria are derived from a filamentous ancestor. For the phylogenetic tree, green = filamentous, black = unicellular.

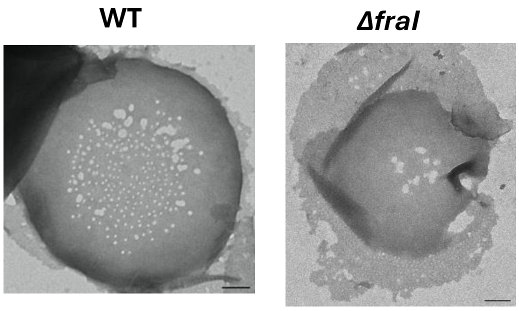

Figure S5. Hormogonia septa of WT and Δ*fraI* with large nanopores. Scale bar – 200 nm

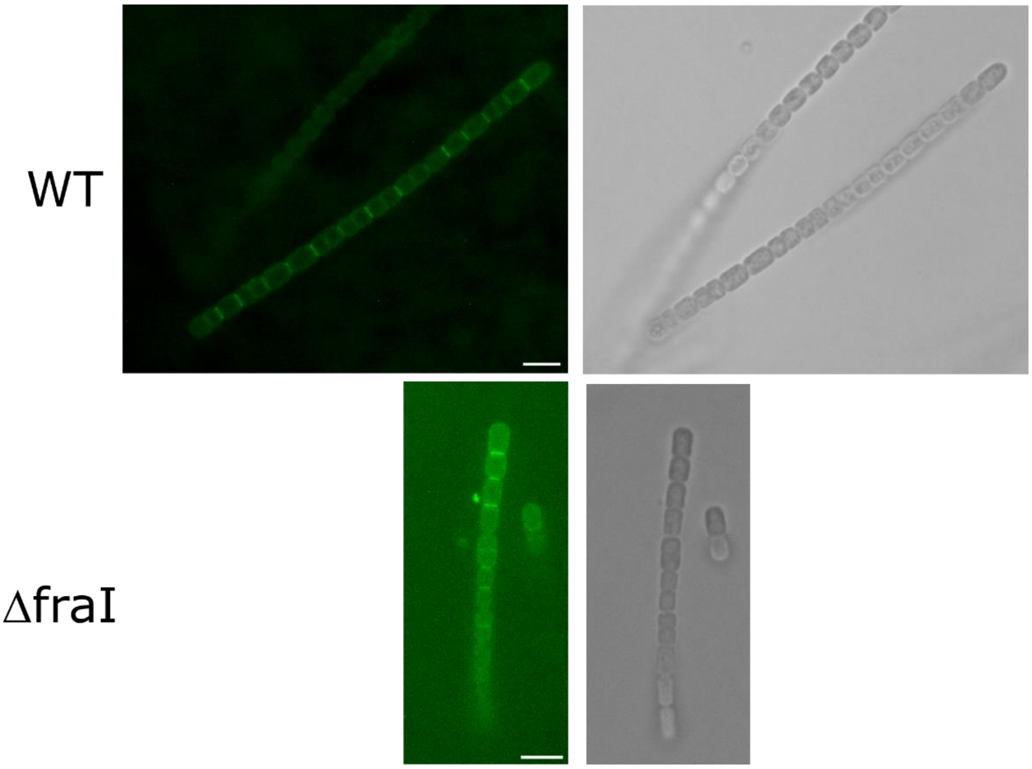

Figure S6 – Vancomycin-FL staining of hormogonia septal peptidoglycan of WT and Δ*fraI*. Scale bar – 5 µm

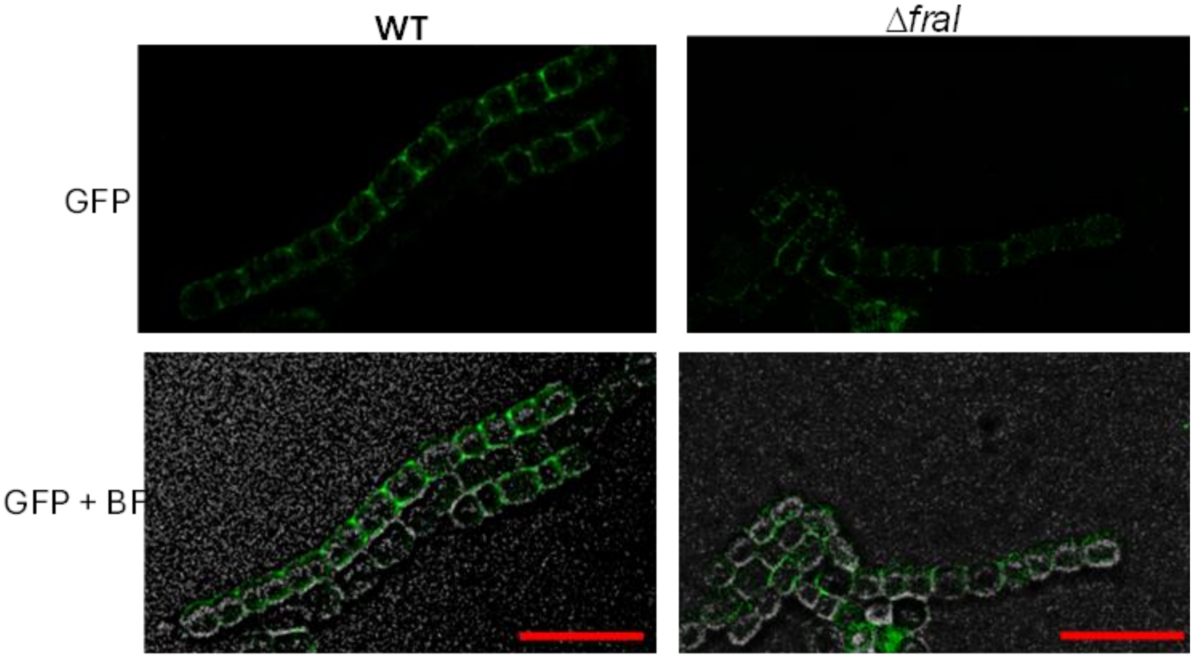

Figure S7 – Immunolocalization of AmiC2 in *Nostoc punctiforme* WT and Δ*fraI* mutant. Scale bar – 10 µm. BF – bright field.

References

1. Cho, Y. W., Gonzales, A., Harwood, T. V., Huynh, J., Hwang, Y., Park, J. S., Trieu, A. Q., Italia, P., Pallipuram, V. K. & Risser, D. D. Dynamic localization of HmpF regulates type IV pilus activity and directional motility in the filamentous cyanobacterium *Nostoc punctiforme*. *Mol Microbiol* **106**, 252–265 (2017).

2. Shih, P. M., Wu, D., Latifi, A., Axen, S. D., Fewer, D. P., Talla, E., Calteau, A., Cai, F., Tandeau De Marsac, N., Rippka, R., Herdman, M., Sivonen, K., Coursin, T., Laurent, T., Goodwin, L., Nolan, M., Davenport, K. W., Han, C. S., *et al.* Improving the coverage of the cyanobacterial phylum using diversity-driven genome sequencing. *Proc Natl Acad Sci U S A* **110**, 1053–1058 (2013).

3. Schirrmeister, B. E., De Vos, J. M., Antonelli, A. & Bagheri, H. C. Evolution of multicellularity coincided with increased diversification of cyanobacteria and the Great Oxidation Event. *PNAS* **110**, 1791–1796 (2013).
